# A Meta-Analysis of Randomized Controlled Trials Comparing the Efficacy and Safety of Hydrophilic Versus Lipophilic Statins in Acute Coronary Syndrome Patients

**DOI:** 10.7759/cureus.68481

**Published:** 2024-09-02

**Authors:** Rofayda M Mohamad, Safiah A Almoayad, Aseel Ahmed A Alanmy, Mohammed Abdullah S Alzahrani, Saeed Hassan S Alshahrani, Bandar Eid H Alharbi, Nahal Hassan A Hassan, Mohammed Khalid A Baqays, Shuruq Talea B Asiri, Fatema Jasim M Meftah, Abdulrahman Ayed R Alharthi, Saleha Mohammed H Ayoub, Maali Hamdan S Alharbi

**Affiliations:** 1 Department of Preventive Medicine, King Salman Armed Forces Hospital in Northwestern Region, Tabuk, SAU; 2 General Practice, King Abdulaziz University Faculty of Medicine, Jeddah, SAU; 3 Medicine and Surgery, King Abdulaziz University Faculty of Medicine, Jeddah, SAU; 4 Medicine and Surgery, Al-Bahah University, Al-Bahah, SAU; 5 Emergency Department, Aseer Hospital, Abha, SAU; 6 College of Medicine, Qassim University, Qassim, SAU; 7 Emergency Department, Prince Sultan Military Medical City, Almadinah Almunawarah, SAU; 8 General Practice, National Guard Health Affairs, Jeddah, SAU; 9 Faculty of Medicine, Najran University, Najran, SAU; 10 Faculty of Medicine, Zhejiang University, Tubli, BHR; 11 Medicine, King Saud Bin Abdulaziz University for Health Sciences College of Medicine, Jeddah, SAU; 12 Faculty of Medicine, Jazan University, Jazan, SAU; 13 Faculty of Medicine, Qassim University, Unizah, SAU

**Keywords:** myocardial infarction, meta-analysis, lipophilic statins, hydrophilic statins, acute coronary syndrome

## Abstract

Statins differ in their solubility. Some previous studies suggested a difference in clinical efficacy and adverse events between hydrophilic and lipophilic statins. The purpose of this study is to compare the efficacy and safety of hydrophilic and lipophilic statins in patients with acute coronary syndrome. The databases of MEDLINE/PubMed, Cochrane Library, the Web of Science, and Scopus were systemically searched for articles published from inception until the 18th of July 2024. The primary outcome included major adverse cardiac events (MACE), while the secondary outcomes included myocardial infarction (MI), unstable angina (UA), revascularization, stroke, all-cause mortality, cardiovascular deaths, and adverse events. The results were pooled as risk ratio (RR) along with their 95% confidence intervals (CI). Nine studies were included. Hydrophilic statins showed a significantly higher risk of MACE and UA compared to lipophilic statins (RR 1.11 [95% CI 1.02, 1.21] and 1.30 [95% CI 1.04, 1.62]), but subgroup analysis showed a lack of significant difference between statins of similar intensity (1.01 [95% CI 0.86, 1.18] and 0.98 [0.67, 1.45], respectively). Both statins showed comparable results regarding the occurrence of MI (1.18 [95% CI 0.98, 1.40]), revascularization (1.09 [95% CI 0.99, 1.20]), stroke (1.16 [95% CI 0.80, 1.66]), all-cause mortality (1.13 [95% CI 0.92, 1.38]), cardiovascular deaths (1.14 [95% CI 0.76, 1.72]), adverse events leading to discontinuation (1.03 [95% CI 0.56, 1.90]), increased alanine aminotransferase (0.61 [95% CI 0.32, 1.16]), increased creatine kinase (0.90 [95% CI 0.30, 2.72]), and increased serum creatinine (1.03 [95% CI 0.49, 2.19]). The efficacy and safety of hydrophilic and lipophilic statins are comparable when the cholesterol-lowering intensity of statins is similar. This suggests that intensity, rather than the lipophilicity of the statin, plays a more important role in the secondary prevention of MACE and individual adverse events.

## Introduction and background

Statins, also known as 3-hydroxy-3-methylglutarylcoenzyme A (HMG-CoA) reductase inhibitors, play a crucial role in the treatment of patients with coronary heart disease, mainly by lowering low-density lipoprotein (LDL) cholesterol levels [1]. In addition, the American College of Cardiology (ACC) and the European Society of Cardiology (ESC) have recommended the use of statins for secondary prevention in patients who experienced acute coronary syndrome (ACS) [2,3].

The main action of statins is the inhibition of the enzyme HMG-CoA reductase that catalyzes the conversion of HMG-CoA to mevalonate; then mevalonate enters the synthesis of cholesterol. Thereby, statins inhibit the synthesis of cholesterol, resulting in enhanced clearance of LDL from the circulation. Meanwhile, statins can exert many pleiotropic effects on several tissues as mevalonic acid is the precursor of numerous metabolites. These various effects include anti-inflammatory and antioxidant activities, improved endothelial function, as well as delayed progression of atherosclerotic plaques [4-7].

Statins differ considerably in their solubility and can be classified as hydrophilic (e.g., simvastatin, fluvastatin, lovastatin, pitavastatin, and atorvastatin) and lipophilic (pravastatin and rosuvastatin) [1]. Solubility can affect the pharmacokinetics and effects of statins. Lipid solubility facilitates the passage of statins into the membranes where they interact with the HMG-CoA reductase enzyme, whereas hydrophilic statins need protein transporters in order to enter the cell to reach their target. Moreover, lipophilic statins are widely distributed to different tissues [8]. 

Some previous studies suggested that hydrophilic statins may exhibit better outcomes compared to lipophilic following myocardial ischemia [9-12]. A recent literature review and a meta-analysis have discussed the differences between lipophilic and hydrophilic statins in patients with coronary heart disease [1,13]. However, evidence is still lacking regarding the comparative efficacy or safety of one class of statins over the other in the instances of secondary prevention following ACS. Therefore, the present meta-analysis aimed to compare the efficacy and safety of hydrophilic and lipophilic statins in patients with acute coronary syndrome.

## Review

Methods

Methodology

The conduction of this study followed the principles of the Cochrane Handbook for Systematic Reviews of Interventions, version 6 and it is reported in accordance with the Preferred Reporting Items for Systematic Reviews and Meta-Analyses (PRISMA) [14].

Eligibility Criteria for the Included Studies

Types of studies: Randomized controlled clinical trials (RCTs) that were published in English, from inception till the 18th of July 2024.

Participants: Patients diagnosed with ACS who received statins.

Intervention and comparison: Direct comparison between hydrophilic and lipophilic statins.

Exclusion criteria: We excluded conference abstracts, duplicate records, observational studies, non-randomized studies, single-arm studies, review articles, commentaries, editorials, clinical guidelines, as well as studies comparing between statins of the same class only (i.e., both are hydrophilic, or both are lipophilic).

Search Strategy 

An online search was carried out on the databases of MEDLINE/PubMed, Cochrane Library, Web of Science, and Scopus. No search filters were used. The search terms for Medline/PubMed included ("myocardial infarction" OR ("Myocardial Infarction"[Mesh]) OR ("Acute Coronary Syndrome"[Mesh])) AND (("lipophilic statin") OR atorvastatin OR simvastatin OR fluvastatin OR pitavastatin OR lovastatin) AND (("hydrophilic statin") OR rosuvastatin OR pravastatin). The Polyglot Search Translator from Systematic Review Accelerator (SRA) [15], Bond University, was used for the formulation of the search terms of the four databases.

Selection of Studies

An online search was carried out by two independent reviewers, who also independently performed screening of the titles and abstracts as well as evaluation of the full text of retrieved records. Discrepancies in decisions between the two reviewers were refereed by consulting the third reviewer.

Data Extraction 

Extracted data included (a) the study design, country, eligibility criteria, sample size, and follow-up; (b), patients' characteristics (e.g., age and sex); (c) the types of statins and their doses; and (d) the outcomes: clinical efficacy and potential adverse events.

Measured Outcomes

Primary outcome: The clinical effectiveness of statins in the secondary prevention of major adverse cardiac events (MACE), including myocardial infarction, unstable angina, revascularization, and death.

Secondary outcomes: The adverse events of statins, including laboratory abnormalities of liver enzymes, creatine phosphokinase, and serum creatinine as well as statin-associated muscle symptoms (SAMS) and novel diagnosis of diabetes mellitus (DM).

Assessment of the Risk of Bias in Included Studies

The risk of bias (ROB) was assessed using the ROB2 tool for randomized clinical trials [16]. The tool encompasses five main domains to assess the risk of bias in the processes of randomization, adherence to the assigned treatment, missed data, measurement of the outcome, and reporting of the outcomes. The overall ROB was identified by selecting the highest level of ROB in the five domains.

Data Synthesis

Pooling of the results was performed with the R statistical language (version 4.4.0; R Foundation for Statistical Computing, Vienna, Austria)) [17], using the packages meta (version 7.0.0) and dmetar (version 0.1.0) [18,19]. The risk ratio (RR) was calculated for the risk of developing the outcomes. In the case of significant heterogeneity (a p-value from the Cochran Chi-square test <0.1 and/or the I2 index ≥50%), a random-effects model was used for pooling the studies’ results. Otherwise, a fixed-effect model was used [20]. A p-value below 0.05 was used to interpret statistical significance of the statistical tests comparing the two statin classes. As the number of included studies was less than 10 trials, publication bias was not tested. Some of the included studies compared statins of different intensities, while others compared statins of the same cholesterol-lowering intensity. Therefore, we divided the studies into two subgroups according to the use of statins with different or similar intensities, based on the classification detailed in the AHA/ACC/AACVPR/AAPA/ABC/ACPM/ADA/AGS/APhA/ASPC/NLA/PCNA guideline for the management of blood cholesterol [2].

Results of literature search and study selection

**Figure 1:**
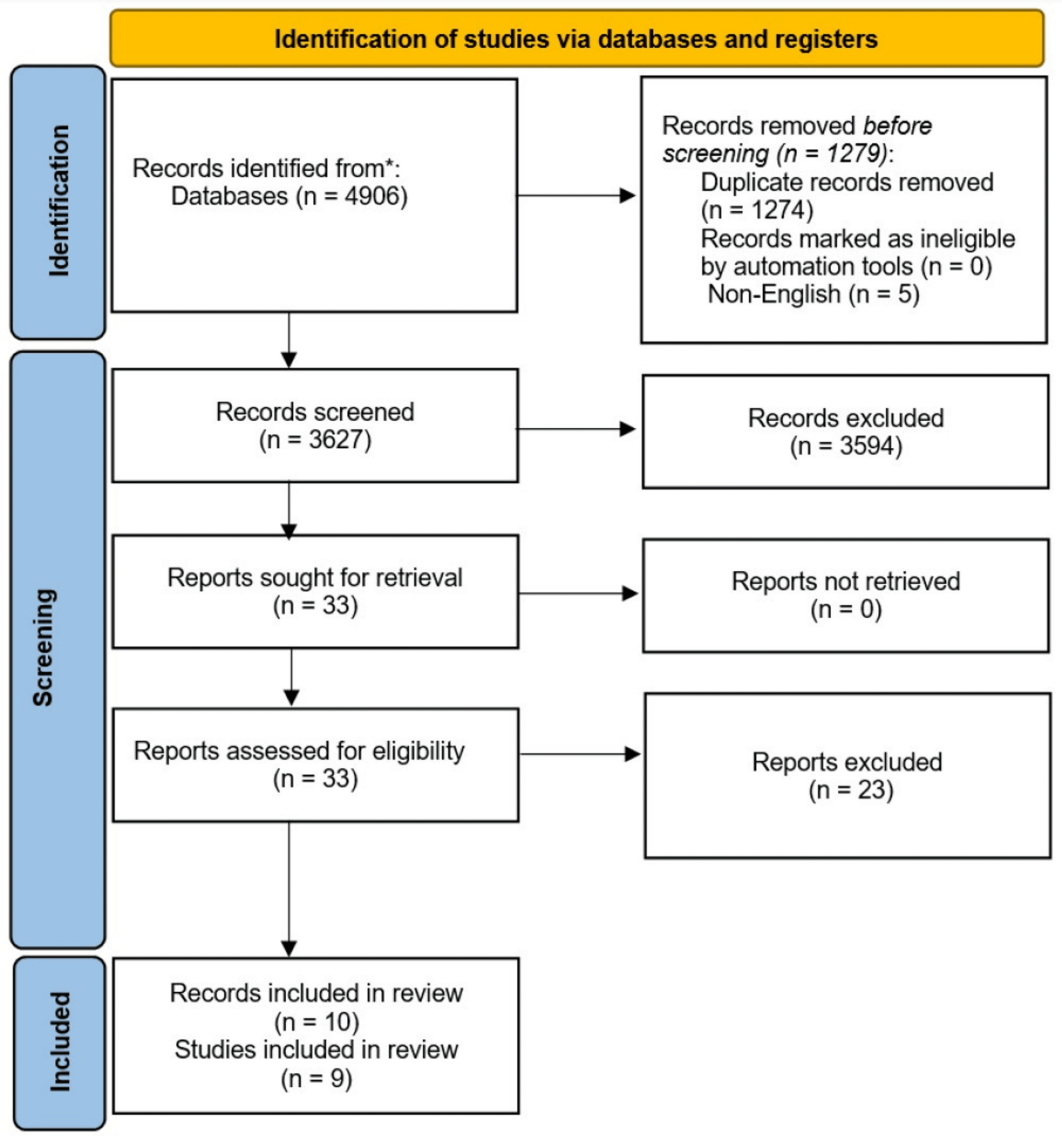
Preferred Reporting Items for Systematic Reviews and Meta-Analyses (PRISMA) flow chart diagram for the results of literature search and study selection

The search strategy produced 4906 records, out of which 1274 were duplicates and five were not published in English. The remaining 3627 records underwent screening of their titles and abstracts, and 3594 records were excluded. The full texts of the remaining 33 records were sought, obtained and assessed for inclusion in this meta-analysis. Twenty-three studies were excluded. Among the 10 eligible records, two records were from the same study. Ultimately, 10 records that come from nine trials were included in this meta-analysis (Figure 1) [10-12,21-27].

Basic Characteristics of the Included Studies

The nine studies included a total of 13,187 participants, with 6,668 randomized into hydrophilic statins and 6,519 assigned into lipophilic statins. All studies were randomized controlled clinical trials. Two studies were conducted in Japan [10,24], and one study in the United Kingdom [22], China [25], Italy [26], and South Korea [27]; the remaining studies were conducted in more than one country [11,12,23]. The follow-up duration varied widely from three months up to 36 months, with a median follow-up duration of 12 months (Table 1).

**Table 1:** Characteristics of the included studies (n = 9) NR: not recorded

Study	Location	Time span	Follow-up (months)
Sakamoto et al. 2007 [10]	Japan	Feb 2002 to Sep 2004	24
Hall et al. 2009 [22]	United Kingdom	May 2005 to Mar 2007	12
Murphy et al. 2009 [23]	8 countries	Nov 2000 to Dec 2001	24
Lablanche et al. 2010 [11]	11 countries	Jan 2006 to Jun 2007	3
Pitt et al. 2012 [12]	United States, Costa Rica, Panama	Dec 2003 to Aug 2007	3
Izawa et al. 2015 [24]	Japan	NR	24
He et al. 2020 [25]	China	Jul 2016 to Dec 2017	6
Toso et al. 2020 [26]	Italy	May 2013 to Sep 2016	12
Lee et al. 2023 [27]	South Korea	Sep 2016 to Nov 2019	36

The sample size also widely varied among the studies, ranging from 30 patients per group and up to 2204 per group. As regards the hydrophilic statins, pravastatin (9-40 mg) was used in three studies [10,23,24], while the other studies used rosuvastatin (10-20 mg). The used lipophilic statins included atorvastatin 9.4-80 mg [10-12,24-27], fluvastatin 26.8 mg [10], and simvastatin 20-40 mg [22,25]. The doses and intensity of statins varied between the studies and even within the same study. The average age in most studies was 60 years, but two studies reported a mean age below 60 [12,23]. Male patients were prevalent in most studies, but one study reported a nearly equal male-to-female ratio [25]. Wide variations among the studies were also observed in the prevalence of hypertension, diabetes mellitus, and smoking (Table 2).

**Table 2:** Summary of baseline criteria in the included studies (n = 9) #: median (interquartile range); DM: diabetes mellitus; H: High intensity; HTN: hypertension; L: low intensity; M: moderate intensity; M-H: Moderate to high intensity; MI: myocardial infarction; N: Number; NR: not recorded; SD: standard deviation

Study	Arm	N	Statin type & dose (mg)	Intensity	Age (years) Mean (SD)	Male %	Previous MI %	HTN %	DM %	Smoking %
Sakamoto et al. 2007 [10]	H	110	Pravastatin 9.4	L	64 (11)	80	5	64	41	50
	L	131	Atorvastatin 9.3 Fluvastatin 26.8	M L	63 (10)	79	3	63	30	51
Hall et al. 2009 [22]	H	633	Rosuvastatin 10	M	62 (12)	79	12	39	12	36
	L	630	Simvastatin 40	M	63 (11)	80	14	39	12	39
Murphy et al. 2009 [23]	H	2063	Pravastatin 40	M	58 (11)	78	19	49	18	37
	L	2099	Atorvastatin 80	H	58 (11)	78	18	51	18	36
Lablanche et al. 2010 [11]	H	437	Rosuvastatin 20	H	60 (11)	74	9	54	19	63
	L	450	Atorvastatin 80	H	59 (12)	76	8	51	18	70
Pitt et al. 2012 [12]	H	277 270	Rosuvastatin 20 Rosuvastatin 40	H	53 (9) 53 (9)	75 74	11 14	52 51	12 13	14 16
	L	278	Atorvastatin 80	H	53 (9)	79	10	50	17	18
Izawa et al. 2015 [24]	H	253	Pravastatin 10-20	L	66 (12)	81	NR	NR	NR	NR
	L	255	Atorvastatin 10-20	M	66 (11)	80	NR	NR	NR	NR
He et al. 2020 [25]	H	32 34	Rosuvastatin 10 Rosuvastatin 20	M H	68 (8) 67 (7)	53 47	NR	25 32	28 29	53 47
	L	33 31 30 32	Atorvastatin 40 Atorvastatin 80 Simvastatin 20 Simvastatin 40	H H M M	69 (8) 69 (7) 70 (8) 66 (9)	55 55 53 50	NR	27 32 30 28	24 36 23 31	49 48 50 47
Toso et al. 2020 [26]	H	355	Rosuvastatin 20	H	69 (58–78)	67	NR	60	27	29
	L	354	Atorvastatin 40	H	68 (58–76)	65	NR	56	24	28
Lee et al. 2023 [27]	H	2204	Rosuvastatin 10-20	M-H	65 (10)	73	NR	68	33	13
	L	2196	Atorvastatin 20-40	M-H	65 (10)	71	NR	66	34	14

Assessment of the Risk of Bias in the Included Studies

The ROB was assessed using the ROB2 tool. The ROB regarding the process of randomization showed some concerns in six studies [10,12,23-25,27], due to a lack of details about the generation of the random sequence [10,12,23,24], allocation concealment [10,12,24,25,27], and/or unequal baseline characteristics between the two groups [23]. The risk of deviations from intended interventions showed some concerns in five studies [12,22,24,25,27], due to non-blinding of participants/carers [12,22,24,27], or the lack of information about the use of an appropriate analysis used to estimate the effect of assignment to intervention [25]. Meanwhile, two studies showed high risk due to non-blinding of participants/carers besides the lack of information about proper testing for assignment to the intervention [10,26]. One study only presented a high risk of missed outcome data due to the lack of information [10]. The ROB was low in all trials in the domain of measurement of the outcome. As regards the domain of selective reporting, three studies presented some concerns due to the non-availability of a pre-specified protocol or analysis plan to compare with the study methods (Table 3) [10,22,25].

**Table 3:** The risk of bias assessment for the included trials based on the ROB2 tool (n = 9) D1: Randomization process; D2: Deviations from intended interventions; D3: Missing outcome data; D4: Measurement of the outcome; D5: Selection of the reported result

Study	D1	D2	D3	D4	D5	Overall
Sakamoto et al. 2007 [10]	Some concerns	High	High	Low	Some concerns	High
Hall et al. 2009 [22]	Low	Some concerns	Low	Low	Some concerns	Some concerns
Murphy et al. 2009 [23]	Some concerns	Low	Low	Low	Low	Some concerns
Lablanche et al. 2010 [11]	Low	Low	Low	Low	Low	Low
Pitt et al. 2012 [12]	Some concerns	Some concerns	Low	Low	Low	Some concerns
Izawa et al. 2015 [24]	Some concerns	Some concerns	Low	Low	Low	Some concerns
He et al. 2020 [25]	Some concerns	Some concerns	Low	Low	Some concerns	Some concerns
Toso et al. 2020 [26]	Low	High	Low	Low	Low	High
Lee et al. 2023 [27]	Some concerns	Some concerns	Low	Low	Low	Some concerns

Results of meta-analysis

MACE

**Figure 2:**
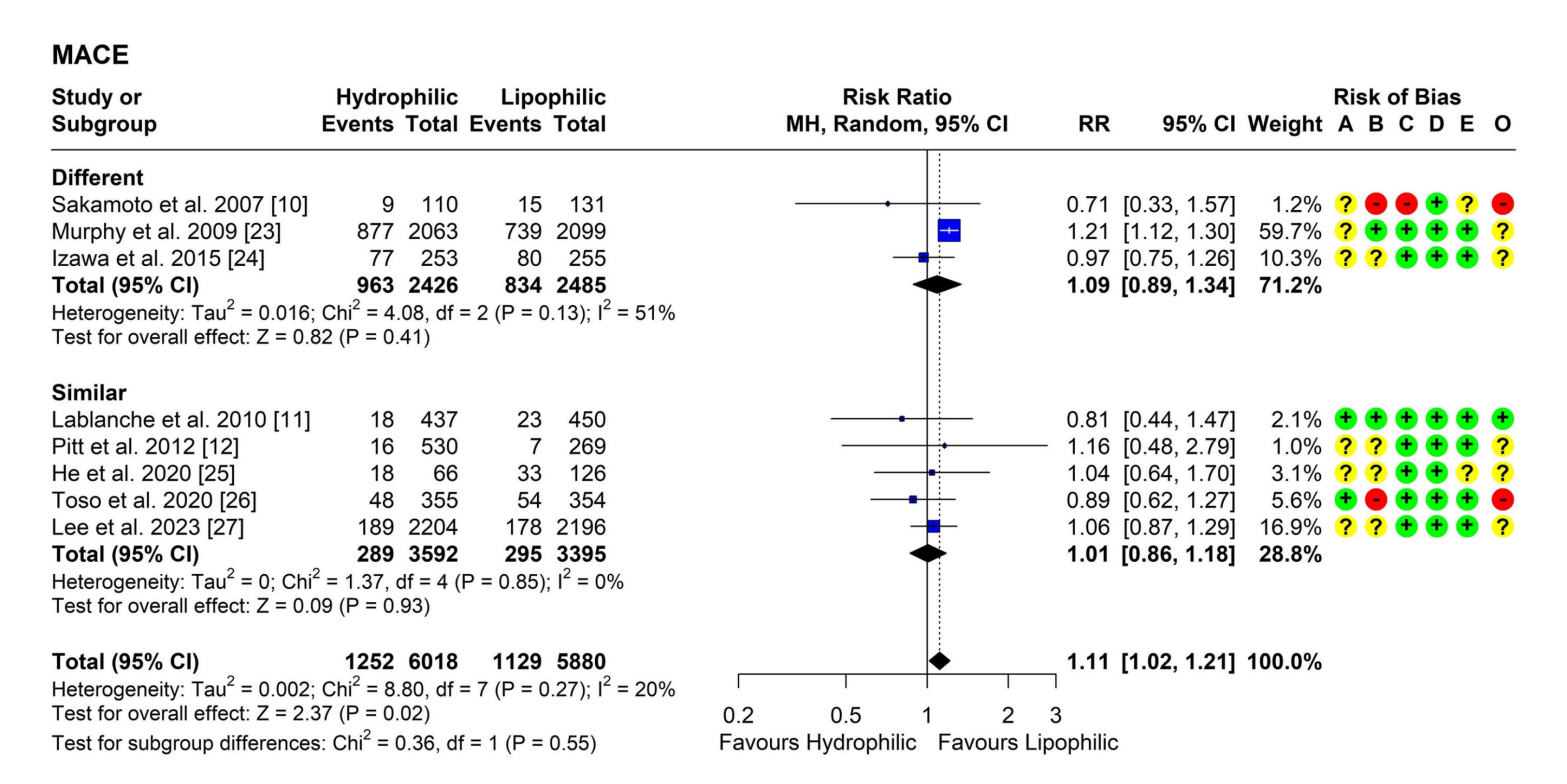
Forest plot showing pooling of the studies’ findings regarding the occurrence of major adverse cardiac events (MACE) CI: Confidence interval; RR: risk ratio; A: Randomization process; B: Deviations from intended interventions; C: Missing outcome data; D: Measurement of the outcome; E: Selection of the reported result; O: Overall risk of bias.

Eight studies reported on the outcome of MACE after the treatment in both groups [10-12,23-27]. Heterogeneity testing for subgroups was significant, so the random effects model was used to pool the results. The pooled RR [95% CI] was 1.11 [1.02, 1.21], P = 0.02 (Figure 2). No outliers were detected but leave-one-out analysis suggested that the study by Murphy et al. may be influential as its omission reduced the I2 index to 0% and the P-value became non-significant [23]. Subgroup analysis showed pooled RRs above one, but non-significant (P > 0.05). Testing for subgroup differences was also non-significant (P = 0.55) (Figure 2).

Myocardial Infarction

**Figure 3:**
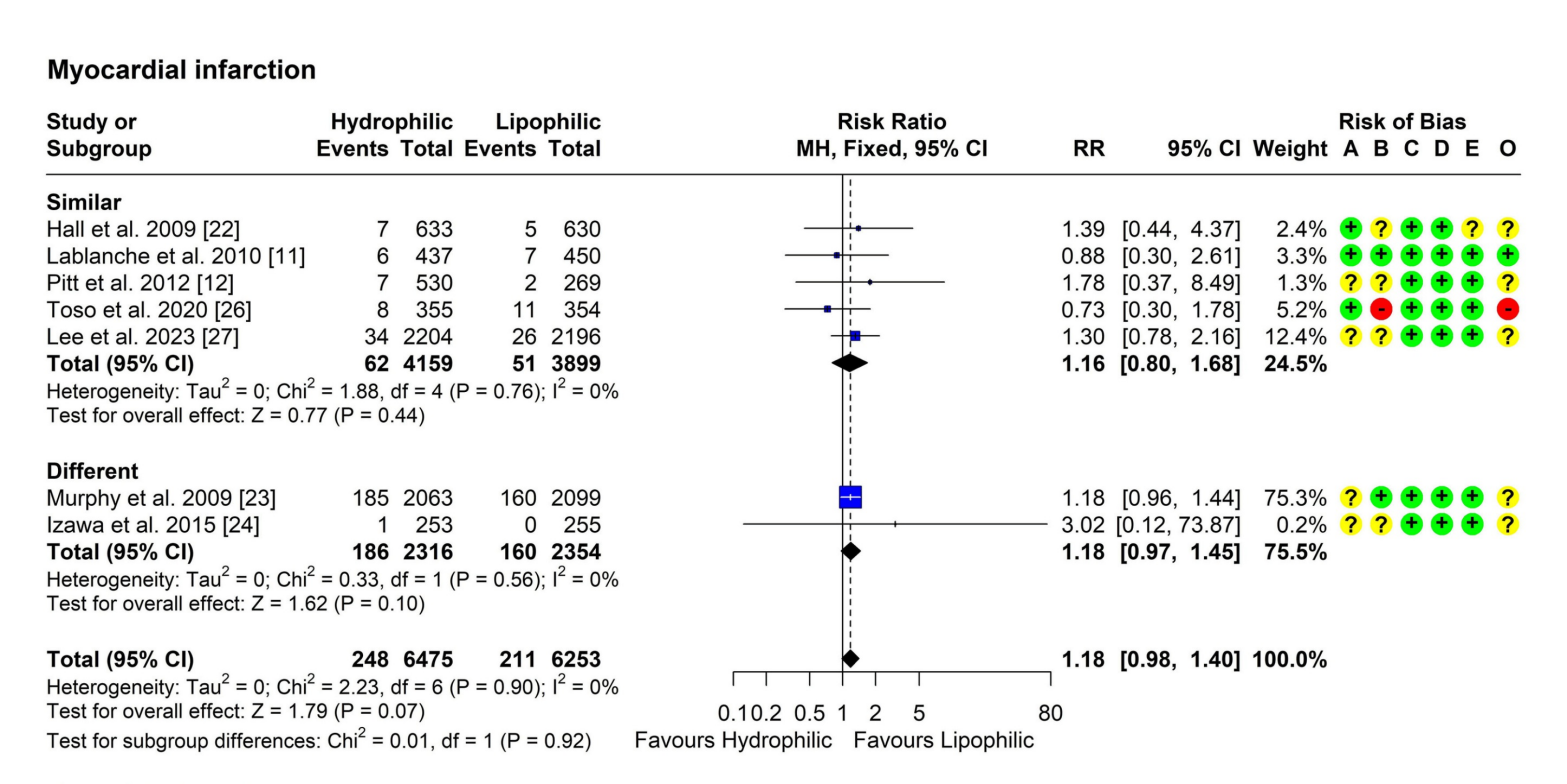
Forest plot showing pooling of the studies’ findings regarding the occurrence of myocardial infarction CI: Confidence interval; RR: risk ratio; A: Randomization process; B: Deviations from intended interventions; C: Missing outcome data; D: Measurement of the outcome; E: Selection of the reported result; O: Overall risk of bias.

Seven studies reported on the outcome of subsequent myocardial infarction in both groups [11,12,22-24,26,27]. Heterogeneity testing was not significant (Chi² = 2.23, P = 0.898, I² = 0%), so the fixed-effect model was used to pool the results. The pooled RR [95% CI] was 1.18 [0.98, 1.40], P = 0.074 (Figure 3). No outliers were detected, and leave-one-out analysis did not suggest the presence of influential studies. Subgroup analysis showed similar results, with no between-group difference (P = 0.92) (Figure 3).

Unstable Angina With Hospitalization 

**Figure 4:**
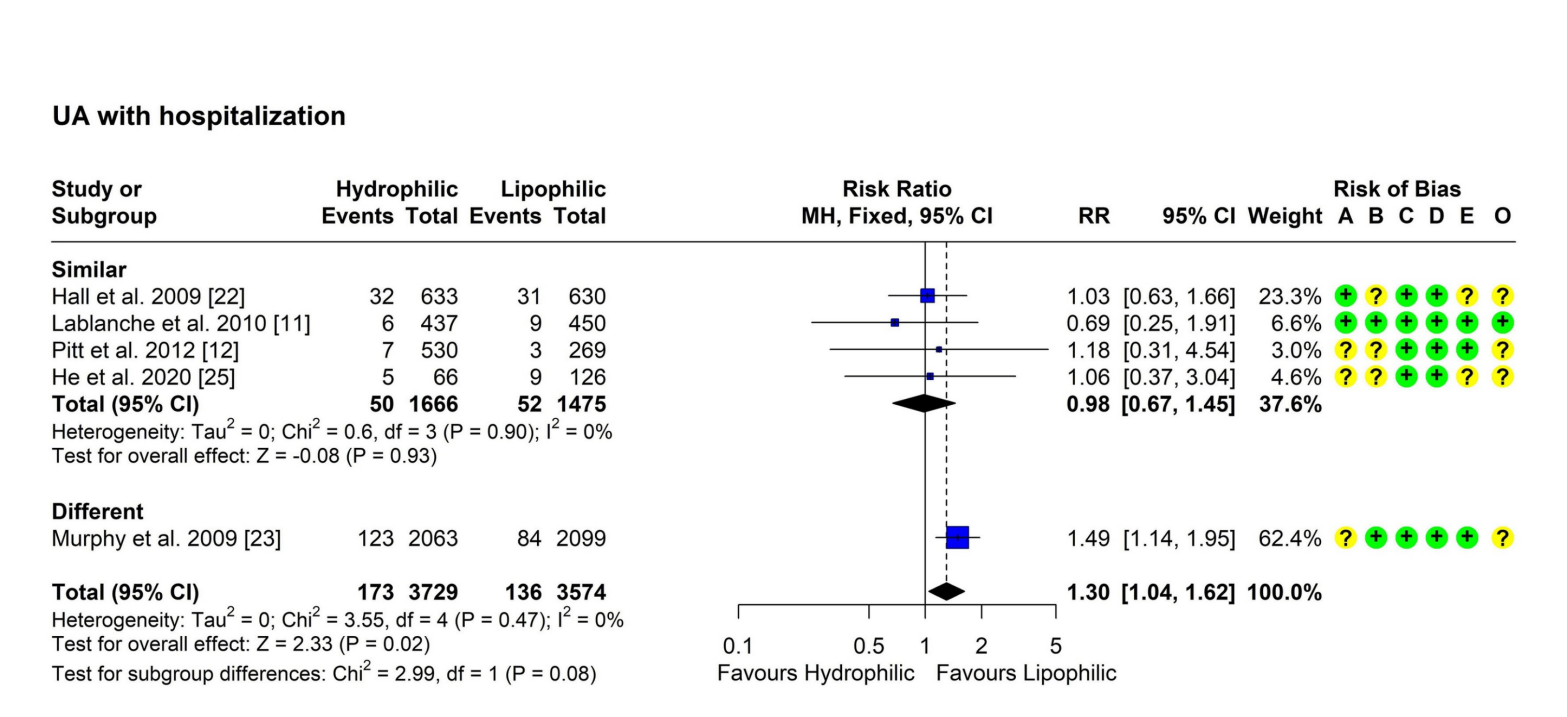
Forest plot showing pooling of the studies’ findings regarding the occurrence of unstable angina with hospitalization CI: Confidence interval; RR: risk ratio; A: Randomization process; B: Deviations from intended interventions; C: Missing outcome data; D: Measurement of the outcome; E: Selection of the reported result; O: Overall risk of bias.

Five studies reported the rate of unstable angina with hospitalization in both groups [11,12,22,23,25]. Heterogeneity testing was non-significant (Chi² = 3.55, P = 0.471, I² = 0%), so the fixed-effect model was used to pool the results. The pooled RR [95% CI] was 1.30 [1.04, 1.62], P = 0.020 (Figure 4). No outliers were detected but leave-one-out analysis suggested that the study by Murphy et al. may be influential as its omission reduced the I2 index to 0% and the p-value became non-significant [23]. Subgroup analysis showed a comparable effect of the two types of statins in studies using similar intensity statins, with no significant difference (P = 0.93) (Figure 4).

Revascularization 

**Figure 5:**
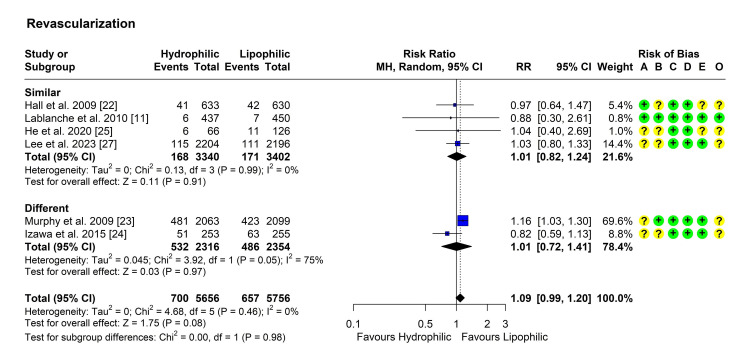
Forest plot showing pooling of the studies’ findings regarding the occurrence of unstable angina with hospitalization CI: Confidence interval; RR: risk ratio; A: Randomization process; B: Deviations from intended interventions; C: Missing outcome data; D: Measurement of the outcome; E: Selection of the reported result; O: Overall risk of bias.

Six studies reported the incidence of revascularization in both groups [11,22-25,27]. Heterogeneity testing was significant within the different-intensity subgroup, so the random effects model was used to pool the results. The pooled RR [95% CI] was 1.09 [0.99, 1.20], P = 0.08 (Figure 5). No outliers were detected but leave-one-out analysis suggested that the study by Murphy et al. may be influential as its omission reduced the RR [23]. Subgroup analyses showed similar results, with no significant difference between the subgroups (P = 0.98) (Figure 5).

Stroke 

**Figure 6:**
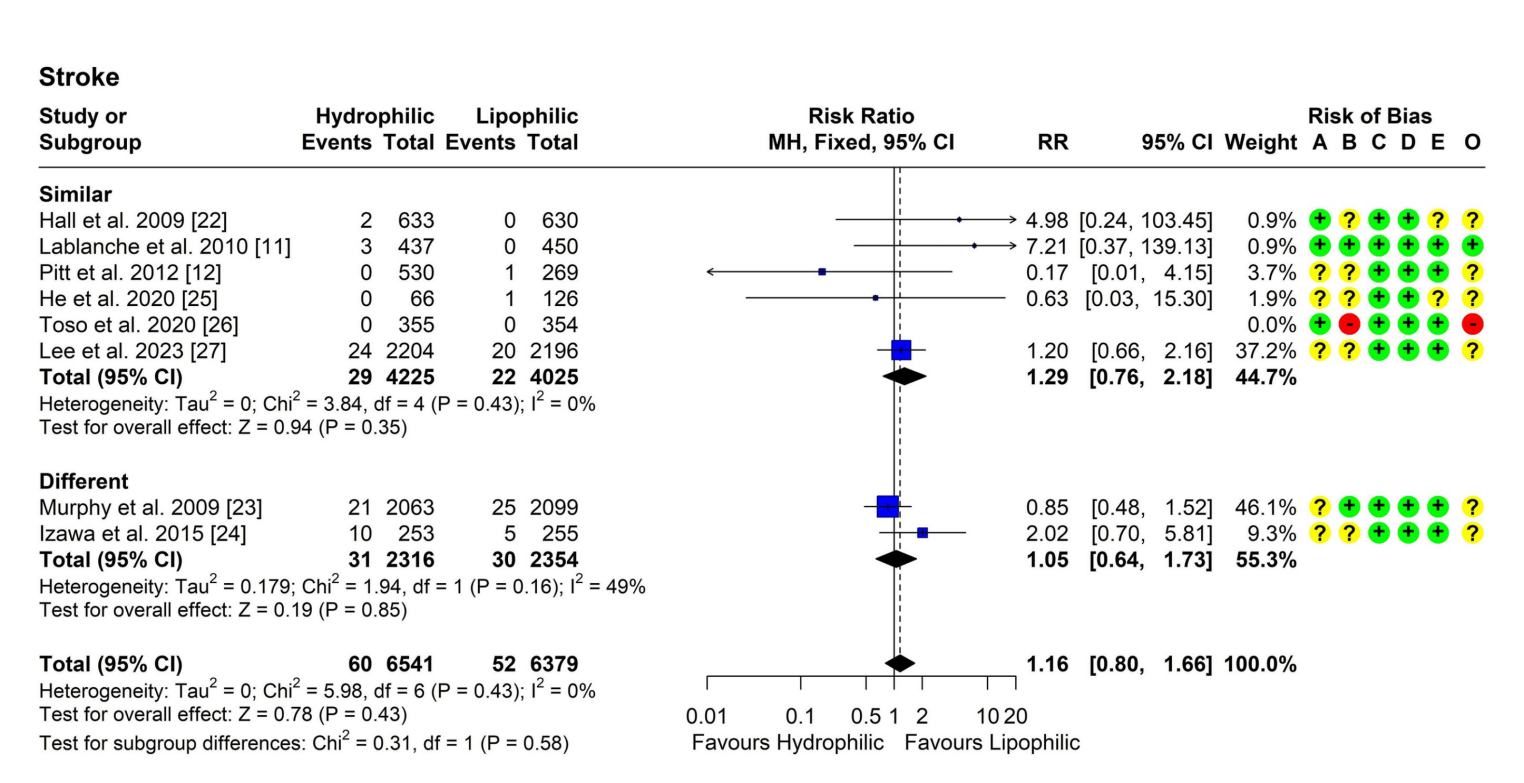
Forest plot showing pooling of the studies’ findings regarding the occurrence of stroke CI: Confidence interval; RR: risk ratio; A: Randomization process; B: Deviations from intended interventions; C: Missing outcome data; D: Measurement of the outcome; E: Selection of the reported result; O: Overall risk of bias.

Seven studies reported the incidence of stroke in both groups [11,12,22-27]. Heterogeneity testing was not significant (Chi² = 5.98, P = 0.425, I² = 0%), so the fixed-effect model was used to pool the results. The pooled RR [95% CI] was 1.16 [0.80, 1.66], P = 0.433 (Figure 6). No outliers or influential studies were detected. Subgroup analyses showed similar results, with no significant difference between the subgroups (P = 0.58) (Figure 6).

All-Cause Mortality

**Figure 7:**
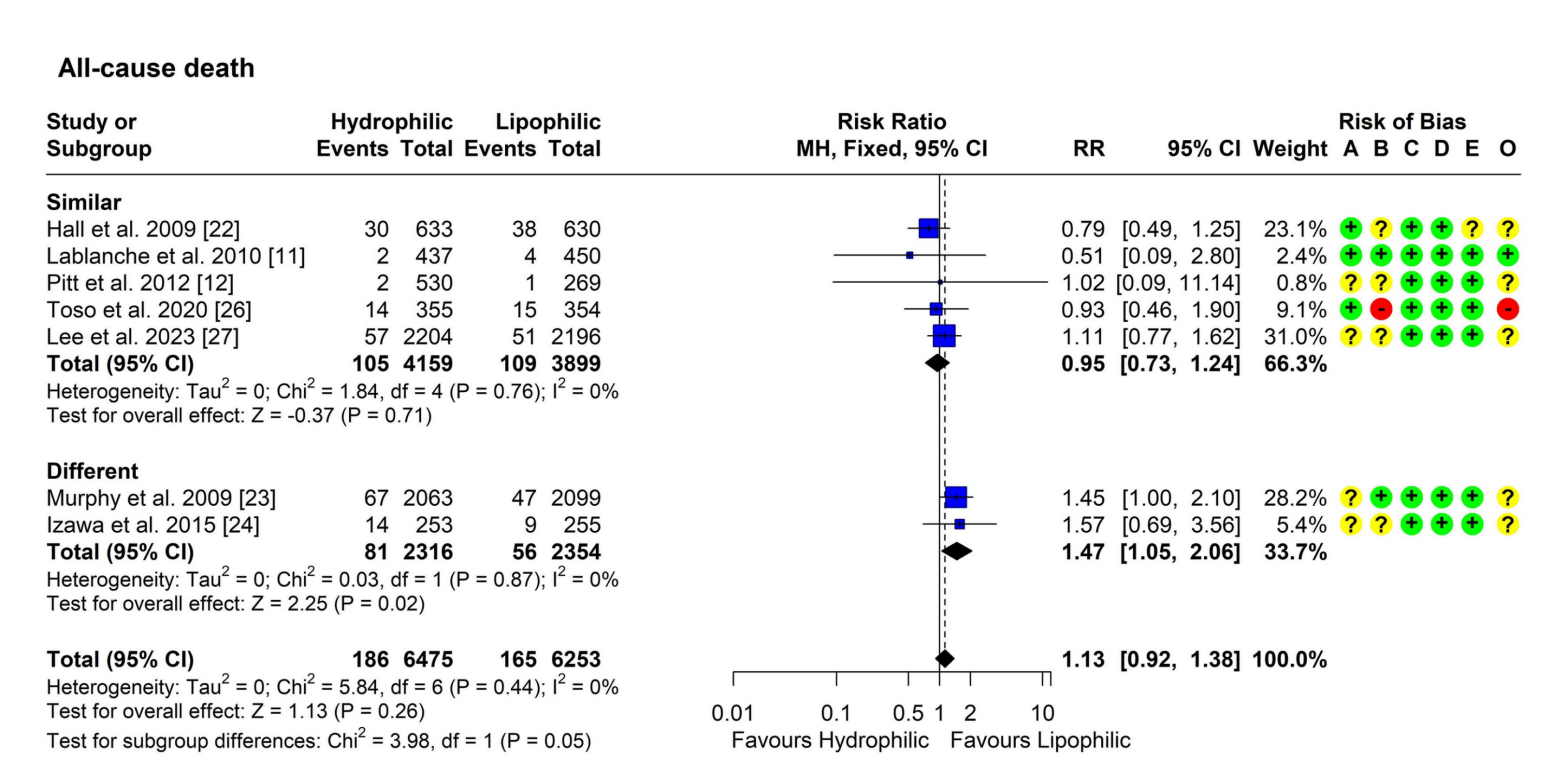
Forest plot showing pooling of the studies’ findings regarding the occurrence of all-cause mortality CI: Confidence interval; RR: risk ratio; A: Randomization process; B: Deviations from intended interventions; C: Missing outcome data; D: Measurement of the outcome; E: Selection of the reported result; O: Overall risk of bias.

Seven studies reported the incidence of all-cause mortality in both groups [11,12,22-24,26,27]. Heterogeneity testing was not significant (Chi² = 5.84, P = 0.441, I² = 0%), so the fixed-effect model was used to pool the results. The pooled RR [95% CI] was 1.13 [0.92, 1.38], P = 0.259 (Figure 7). No outliers or influential studies were detected. Subgroup analysis showed the lack of significant difference in studies using statins with similar intensity (P = 0.71), contrary to studies with different intensities (P = 0.02). Subgroup testing yielded a marginal P-value of 0.05 (Figure 7).

Death Due to Cardiovascular Causes

**Figure 8:**
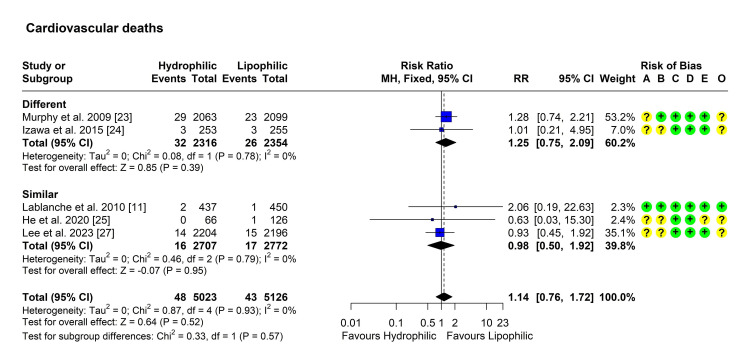
Forest plot showing pooling of the studies’ findings regarding the occurrence of death due to cardiovascular causes CI: Confidence interval; RR: risk ratio; A: Randomization process; B: Deviations from intended interventions; C: Missing outcome data; D: Measurement of the outcome; E: Selection of the reported result; O: Overall risk of bias.

Five studies reported the incidence of cardiovascular deaths in both groups [11,23-25,27]. Heterogeneity testing was not significant (Chi² = 0.87, P = 0.929, I² = 0%), so the fixed-effect model was used to pool the results. The pooled RR [95% CI] was 1.14 [0.76, 1.72], P = 0.524 (Figure 8). No outliers or influential studies were detected. Subgroup testing showed the absence of significant differences (P = 0.57) (Figure 8).

Adverse Events Leading to Discontinuation

Four studies reported the incidence of adverse events resulting in statin discontinuation [11,12,22,24]. Heterogeneity testing was significant (Chi² = 9.42, P = 0.024, I² = 68%), so the random effects model was used to pool the results. The pooled RR [95% CI] was 1.03 [0.56, 1.90], P = 0.913 (Figure 7). No outliers were detected, but the omission of the studies by Hall et al. and Pitt et al. reduced heterogeneity considerably [22,12].

Increased Alanine Aminotransferase (ALT) Level 

**Figure 9:**
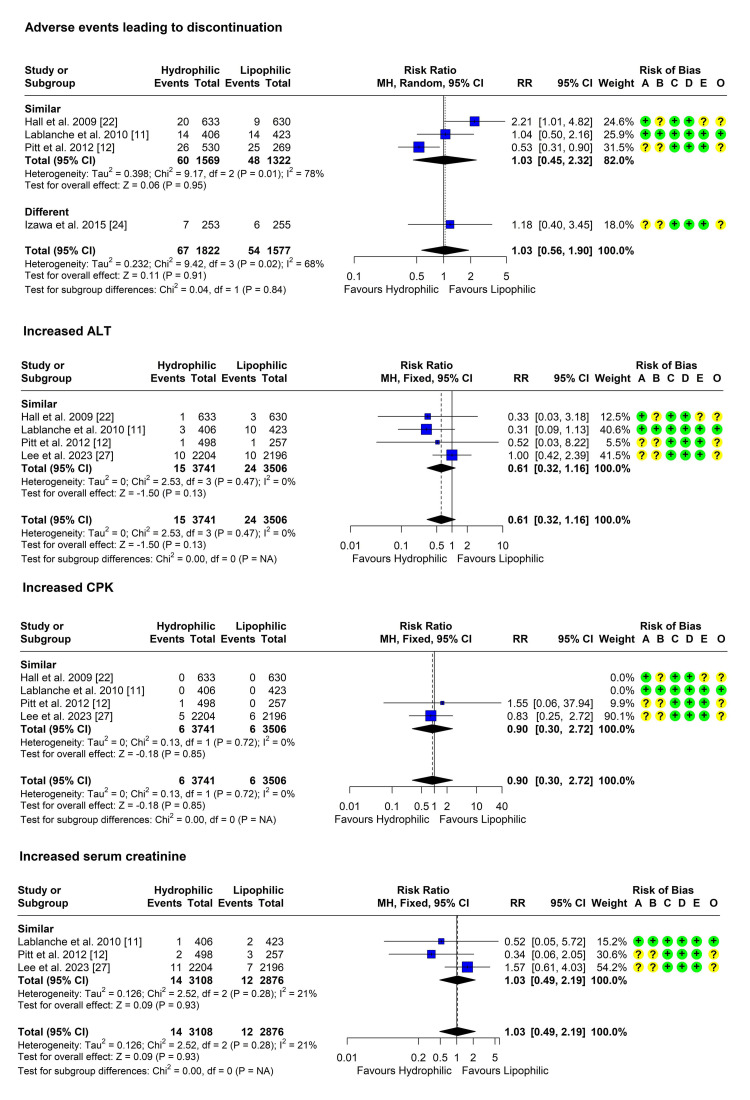
Forest plot showing pooling of the studies’ findings regarding the occurrence of adverse events CI: Confidence interval; RR: risk ratio; A: Randomization process; B: Deviations from intended interventions; C: Missing outcome data; D: Measurement of the outcome; E: Selection of the reported result; O: Overall risk of bias; ALT: Alanine aminotransferase; CPK: Creatine phosphokinase

Four studies reported the incidence of increased ALT levels [11,12,22,27]. Heterogeneity testing was not significant (Chi² = 2.53, P = 0.470, I² = 0%), so the fixed-effect model was used to pool the results. The pooled RR [95% CI] was 0.61 [0.32, 1.16], P = 0.133 (Figure 9). No outliers or influential studies were detected.

Increased Creatine Phosphokinase (CPK) Level

Four studies reported the incidence of increased CPK levels [11,12,22,27]. Heterogeneity testing was not significant (Chi² = 0.13, P = 0.719, I² = 0%), so the fixed-effect model was used to pool the results. The pooled RR [95% CI] was 0.90 [0.30, 2.72], P = 0.854 (Figure 9). No outliers were detected, but omitting the study by Lee et al. increased the RR above 1 [27].

Increased Serum Creatinine

Three studies reported the incidence of increased creatinine levels [11,12,27]. Heterogeneity testing was not significant (Chi² = 2.52, P = 0.284, I² = 21%), so the fixed-effect model was used to pool the results. The pooled RR [95% CI] was 1.03 [0.49, 2.19], P = 0.932 (Figure 9). No outliers were detected.

Discussion

Summary of the Main Findings

As statins differ in their solubility, previous studies assessed the comparative efficacy and safety of hydrophilic and lipophilic statins [9-12]. This meta-analysis aimed to compare the efficacy and safety of hydrophilic and lipophilic statins in patients with acute coronary syndrome.

The hypothesis underlying the difference in efficacy between the two types of statins depends on the higher ability of lipophilic statins to penetrate cell membranes. Therefore, lipophilic statins can penetrate more readily into extrahepatic tissues [28], and thus they can affect myocardial contraction [9]. In addition, the enzyme targeted to prevent cholesterol synthesis, HMG-CoA reductase, is a membrane-associated protein in the endoplasmic reticulum; thus, the lipophilicity of statins will impact their interaction with the membrane and the inhibition of the enzyme [29].

Meanwhile, besides their lipid-lowering effect, both hydrophilic and lipophilic statins increase the synthesis and release of nitric oxide [30], thereby exerting a cardioprotective effect against ischaemia-reperfusion injury, and decreasing the size of developed infarct [31,32]. The current meta-analysis showed a lack of significant differences between the two types of statins regarding any of the assessed outcomes. Although the overall pooled RR suggested a significantly higher risk of MACE and UA with hydrophilic statins (RR 1.11 [95% CI 1.02, 1.21] and 1.30 [95% CI 1.04, 1.62], respectively), subgroup differences based on intensity negated this difference when statins of similar intensity were compared. The results suggest that the intensity of statins, rather than their solubility, may affect their efficacy.

Another important concern is the safety of statins, particularly due to their long-term administration in patients with coronary heart disease. Lipophilic statins were presumed to carry a higher risk of adverse events, owing to their higher penetration of extrahepatic tissues. However, solid evidence is still lacking in this field [1]. We found that the risk of adverse events was comparable between the two types of statins. 

A recent meta-analysis has compared hydrophilic and lipophilic statins [13], but in a more diverse population of coronary heart disease (including both ACS and chronic heart disease). They reported similarly a lack of significant difference between the two statins regarding the clinical outcomes of efficacy. However, they found that hospitalization due to cardiovascular causes was lower (0.789, 95%Cl: 0.643 to 0.969, P = 0.024) and ALT elevation was higher (2.689, 95%Cl: 1.841 to 3.954, P<0.001) with lipophilic statins compared to hydrophilic statins.

Overall Completeness, Applicability, and Quality of the Evidence

The evidence summarized in the current meta-analysis suggests the comparable efficacy and safety of hydrophilic and lipophilic statins as secondary prevention after ACS. The meta-analysis included studies with large sample sizes, all of which were RCTs that are bound to provide a high grade of evidence. However, the quality of evidence is marred by the potential ROB introduced into the included studies. In addition, the studies showed marked clinical heterogeneity regarding the age, sex, and medical history of patients as well as the intensity of used statins and the length of follow-up. All these factors can affect the results of this meta-analysis.

## Conclusions

The efficacy and safety of hydrophilic and lipophilic statins are comparable when the cholesterol-lowering intensity of statins is similar. This suggests that intensity, rather than the lipophilicity of the statin, plays a more important role in the secondary prevention of MACE and individual adverse events. Future RCTs should focus on comparing statins with the same intensity and for long-term follow-up (above two years) to assess for adverse events arising because of long-term administration.
